# The Palliative-Supportive Care Unit in a Comprehensive Cancer Center as Crossroad for Patients’ Oncological Pathway

**DOI:** 10.1371/journal.pone.0157300

**Published:** 2016-06-22

**Authors:** Sebastiano Mercadante, Claudio Adile, Amanda Caruselli, Patrizia Ferrera, Andrea Costanzi, Paolo Marchetti, Alessandra Casuccio

**Affiliations:** 1 Anesthesia and Intensive Care Unit and Supportive-Palliative Care Unit, La Maddalena Cancer Center, Palermo, Italy; 2 Home Care program, SAMO, Palermo, Italy; 3 Department of Oncology, Hospital Sant’Andrea, University of Rome, Rome, Italy; 4 Department of Experimental Biomedicine and Clinical Neuroscience, University of Palermo, Palermo, Italy; University Campus Bio-Medico, ITALY

## Abstract

**Aim:**

The aim of this study was to assess how an admission to an acute palliative-supportive care unit (APSCU), may influence the therapeutic trajectory of advanced cancer patients.

**Methods:**

A consecutive sample of advanced cancer patients admitted to APCU was assessed. The following parameters were collected: patients demographics, including age, gender, primary diagnosis, marital status, and educational level, performance status and reasons for and kind of admission, data about care-givers, recent anticancer treatments, being on/off treatment or uncertain, the previous care setting, who proposed the admission to APSCU. Physical and psychological symptoms were evaluated at admission and at time of discharge. The use of opioids was also recorded. Hospital staying was also recorded. At time of discharge the parameters were recorded and a follow-up was performed one month after discharge.

**Results:**

314 consecutive patients admitted to the APSCU were surveyed. Pain was the most frequent reason for admission. Changes of ESAS were highly significant, as well as the use of opioids and breakthrough pain medications (p <0.0005). A significant decrease of the number of “on therapy” patients was reported, and concomitantly a significant number of “off-therapy” patients increased. At one month follow-up, 38.9% patients were at home, 19.7% patients were receiving palliative home care, and 1.6% patients were in hospice. 68.5% of patients were still living.

**Conclusion:**

Data of this study suggest that the APSCU may have a relevant role for managing the therapeutic trajectory of advanced cancer patients, limiting the risk of futile and aggressive treatment while providing an appropriate care setting.

## Introduction

Cancer patients may develop relevant symptoms during the course of disease. They spend more time in hospital wards for concurrent complications, uncontrolled symptoms, or to receive aggressive therapies, even in last weeks of life [1]. Other than common symptoms due to disease progression, anti-cancer toxicities represent a serious burden for cancer patients often resulting in unplanned or planned hospital presentations. These aspects require expert advice for facing complex clinical and psychological problems [2]

Palliative care is commonly provided at home, in hospice, or occasionally with mobile team in hospitals, when available. Regrettably, in most countries specialized palliative care is provided only for 2–3 weeks before death [3]In a recent survey, in the last three months before hospice admission, a large amount of patients were admitted to hospital for about five weeks, and about half of patients had received chemotherapy. Most of these patients had a relevant symptom burden and were undertreated. Almost all patients died in hospice within 2–3 weeks, suggesting that hospice admission is only one way for end of life treatments [4]Similarly, it has been reported that in the last months of life most patients spent about 1/3 of this period in hospital, with half of them receiving chemotherapy in the last month of life [5]The mean survival of cancer patients followed by a home palliative care team has been reported to be 6–7 weeks [6]Indeed, many patients receive aggressive oncologic treatments even at the end of life and futile treatments are often administered because the poor knowledge or experience of oncologists in palliative care [1,7–9]. In a prestigious institution in USA, the interval between palliative care referral and death was less than two months [10].This timing for palliative care is inacceptable for patients who commonly present clinical problems early during the course of disease.

Recent evidence strongly suggests to start palliative care in other settings, other than traditional home care and hospice, to intercept cancer patients early in the course of disease, rather than limiting this intervention in the last weeks of life. An early referral to a palliative care team should be optimal to provide immediate symptom relief, treatment of drug-induce toxicities, education, and advice on the future therapeutic pathways [11–16]. In the last years, an increased number of acute palliative-supportive care units (APSCU) in comprehensive cancer centers has been developed. A specialistic team can meet the global needs of cancer patients at any stage of disease to resolve the many physical and psychological problems occurring during both the active treatment or the advanced stage of disease, including the delicate phase of end of life [11–18].Several studies have reported the positive outcomes of APSCUs, that have been shown to provide better outcomes and cost saving than palliative care consultation services [13,19–25].However, the role of an APSCU admission in re-directing the oncologic pathway, other than symptom control, has not well investigated. In other words, can an APSCU admission influence the therapeutic trajectory of advanced cancer patients? This outcome may have a tremendous impact on patients’ care, avoiding further futile and expensive treatments while providing a timely and appropriate intervention. The aim of this study was to assess how an admission to an APSCU in a comprehensive cancer center can influence the therapeutic trajectory of oncological patients and optimize consequently the resources.

## Material and Methods

The institutional review board at the University of Palermo approved the study and written patients’ informed consent was obtained. The study was performed in an APSCU, devoted to research and connected to the University of Palermo. This eight beds unit was established about 15 years ago in a small comprehensive cancer center. The characteristics of this unit have been described elsewhere [17].The goals of this unit are the control of pain and symptoms due to disease, drug-related toxicities, providing advice to other units, and facilitate the transition for care, when indicated.

A consecutive sample of advanced cancer patients who were admitted to an APSCU was prospectively assessed for a period of 10 months. Advanced cancer was considered as locally advanced, recurrent, or metastatic disease for solid cancers, and relapsed or refractory disease for hematologic tumors. All patients underwent comprehensive and continuous symptom assessment and management during their hospital stay at APSCU [17]. At discharge, patients’ situation was reviewed and eventually an oncologic consultation was asked to decide the next therapeutic steps.

### Data Collection

Patients demographics, including age, gender, primary diagnosis, marital status, and educational level were collected. Performance status and reasons for admission were recorded, including pain or opioid-related problems, toxicity from chemotherapy, symptom control, re-evaluation, or end-of-life issues. The kind of admission was also characterized as unplanned or planned admission. Eventual re-admissions and their principal indications, and time from the previous admission, were recorded. Data regarding people living with the patients were gathered. A care giver was considered as a person who spent at least 4 hours/day with the patient. Patients’ and caregivers’ awareness of prognosis was assessed by semi-structured interviews (complete, partial, absent). The following data were also recorded: chemotherapy in the last 30 days, or other anticancer treatments (radiotherapy, surgery, target therapy, hormonal therapy, and so on), on/off treatment or uncertain, that is when the need of oncologic treatment remained to be established and physicians delay taking a decision. It was also recorded the previous care setting, including home, hospital unit, day-hospital, or other hospitals, and who referred the patient to the unit, including home palliative care physicians, oncologists, other units, other hospitals, or general practitioner (GP).

Physical and psychological symptoms were evaluated by Edmonton Symptom Assessment Scale (ESAS) at admission and at time of discharge (or the day before death). ESAS is a self-reported tool assessing the intensity of most common psychological and physical symptoms on a 0 to 10 numerical scale, rated on the average score in the previous 24-hour period. ESAS is a valid and reliable tool for assessing the overall symptom burden, sensible to changes produced by a treatment [26,27].A screening test for history of alcohol dependence (CAGE: cut down, annoy, guilt, eye-opener) was also administered [28].The Memorial delirium Assessment Scale (MDAS) was used to assess the cognitive status of patients. MDAS is a validated tool to quantify the intensity of delirium [29].Analgesic drugs and their doses at admission and discharge were recorded. Hospital staying was also recorded. At time of discharge the outcome and subsequent referral to other care settings (death, home, home care, hospice, oncology), and the pathway of oncologic treatment were re-considered (on/off, uncertain). One month after discharge, patients or their relatives were contacted by phone to gather information on the care setting, if the patients were continuing anticancer therapies, or died.

### Statistical Analysis

Statistical analysis of quantitative and qualitative data, including descriptive statistics, was performed for all items. Continuous data were expressed as mean ± standard deviation (SD), unless otherwise specified. Frequency analysis was performed using the Pearson’s chi-square test and Fisher exact test, as needed. The univariate analysis of variance was used for parametric analysis; the paired samples Student's t-test was used to compare symptom intensity and opioid dosage, respectively, at the different intervals. Data were analyzed by IBM SPSS Software 22 version (IBM Corp., Armonk, NY, USA). All p-values were two-sided and p<0.05 was considered statistically significant.

## Results

314 consecutive cancer patients admitted to the APSCU were surveyed. For the different parameters taken into consideration, data were missed for less than 10% of patients (range 0–20 patients). The characteristics of patients, stage of disease, kind of admission (urgent, readmission etc), information regarding relatives and the caregiver, educational level, indications for admission, active treatments in the last two weeks, and patients’ and caregivers’ awareness of disease are presented in Table 1.

**Table 1:** Characteristics of patients.

**Patients**	**314**	
**Age** (yrs, mean SD)	65.7 (12)	
**Gender**	132 F /182 M	
**Karnofsky** (mean SD)	46 (11.3)	
**Primary tumor**		
Lung	62	
Breast	51	
Genitourinary	49	
Gastrointestinal	47	
Liver	19	
Pancreas	18	
Head-neck	13	
Unknown	12	
Hematologic disease	19	
Other	24	
**Stage of disease**		
Locally advanced	49 (15.6%)	
Metastatic	238 (75.8%)	
No evidence of disease	27 (8.6%)	
**People living with the patients**		
Alone	27 (8.6%)	
Partner	123 (39.2%)	
Partener and/or sons/daughters	287 (91.4%)	
Nursing home	4 (1.3%)	
Presence of care-giver	265 (84.4%)	
**Education**		
No school	12 (3.8%)	
Primary	103 (32.8%)	
Secondary school	98 (31.2%)	
High level	61 (19.4%)	
Degree	40 (12.8%)	
**Disease awareness**	*Patient*	*Care-giver*
Complete	165 (52.5%)	269 (85.7%)
Partial	120 (38.2%)	36 (11.5%)
Absent	29 (9.2%)	9 (2.8%)
**Admission**		
Planned admission	246 (78.3%)	
Unplanned admission	55 (17.5%)	
Transfer from other units	13 (4.2%)	
**Indications for admission (multiple choice)**		
Uncontrolled pain	231 (73.6%)	
Opiod-related toxicity	61 (19.4%)	
Anticancer toxicity	58 (18.5%)	
Other symptoms	182 (58%)	
End of life care	25 (8%)	
**Treatments in the previous 30 days**		
Chemotherapy	97 (30.9%)	
Chemotherapy + target therapy	8 (2.5%)	
Chemotherapy + radiotherapy	3 (1%)	
Target therapy	17 (5.4%)	
Radiotherapy	6 (1.9%)	
Hormonal therapy	12 (3.8%)	
Hormonal + target therapy	2 (0.6%)	
Surgery	29 (9.2%)	

Most admissions were planned, but a consistent number of patients admitted on emergency (generally from the oncological day-hospital) or transferred from other hospital units. The mean hospital stay was 6.9 days (SD 6.3). Twelve patients (3.8%) died in the unit. A significant number of patients were assigned to home palliative care or hospice admission at time of discharge.

Data regarding on how the patient was considered for treatment purposes (on/off, or uncertain), the setting where patients were previously cared, who referred the patient for admission, and clinical pathways suggested at discharge from the unit, are presented in Table 2.

**Table 2:** Data regarding the previous disease-oriented treatment, the setting where patients were previously cared and who referred the patient at admission, and care setting, physicians to refer patients and clinical pathways suggested at discharge from the unit. APSCU = acute palliative-supportive care unit.

**Proposal for referral**			
GP	6 (1.9%)		
	192 (61.2%)		
	6 (1.9%)		
	46 (14.6%)		
	24 (7.6%)		
	26 (8.3%)		
	14 (4.5%)		
**Care setting**			
	*at admission*	*proposed at discharge*	*P*
Home	256 (81.5%)	203 (64.6%)	<0.0005
Home palliative care	28 (8.9%)	92 (29.3%)	<0.0005
Hospice	0	12 (3.8%)	<0.0005
Oncology	4 (1.3%)		
Other units	14 (4.5%)		
Emergency	4 (1.3%)		
Other hospitals	8 (2.5%)	7 (2.3%)	1.0
**Disease-oriented treatment**			
	*at admission*	*proposed at discharge*	*P*
On	150 (47.8%)	105 (33.4%)	<0.0005
Off	73 (23.2%)	103 (32.8%)	0.0043
Uncertain	65 (20.7%)	74 (23.6%)	0.441
None	14 (4.5%)	18 (5.7%)	0.586
Follow-up	12 (3.8%)	14 (4.5%)	0.842

At discharge a significant decrease of the number of “on therapy” patients was reported, and concomitantly the number of “off-therapy” patients increased. Regarding the possible relationships with the parameters considered, both epidemiological and sociocultural, younger patients, females, and diagnosis of breast cancer were more likely to be “on therapy” (p = 0.029, p = 0.0005, and p = 0.010, respectively). Younger patients and patients who had a high Karnofsky status were more likely to be discharged home (p = 0.021, and p = <0.0005, respectively). No other significant relationships with clinical and sociocultural variables were found.

### Changes in ESAS and Analgesic Drugs

ESAS at time of admission and at discharge (one day before death in patients who died) are presented in Table 3. The difference was highly significant for all the items. At admission 197 patients were receiving opioids (in a rank order: n.38 oxycodone/naloxone, n.23 transdermal fentanyl, n.17 codeine, n.15 morphine, n.14 hydromorphone, n.12 methadone, n.12 tapentadol, and others). At discharge 236 patients were prescribed opioids (in a rank order: n.44 hydromorphone, n.30 transdermal fentanyl, n.28 methadone, n.22 morphine, n.20 oxycodone/naloxone, n.13 transdermal buprenorphine, and others). The difference was significant (p = 0.001). Oral morphine equivalents at admission and at discharge were 190 mg (SD 162), and 215 mg (SD 121), respectively.

**Table 3:** ESAS items at admission and at discharge in patients with complete data.

	N	at admission	at discharge	P
Pain	263	5.26 (2.83)	2.59 (1.98)	0.001
Weakness	263	5.64 (2.82)	3.27 (2.67)	0.001
Nausea	259	1.96 (2.77)	0.90 (1.89)	0.001
Depression	261	3.15 (2.03)	1.69 (2.21)	0.001
Anxiety	260	2.94 (3.06)	1.68 (2.31)	0.001
Drowsiness	260	4.18 (2.70)	2.86 (2.60)	0.001
Dyspnea	261	2.34 (3.14)	1.34 (2.26)	0.001
Insomnia	262	4.44 (3.25)	2.64 (2.82)	0.001
Appetite	260	5.88 (2.31)	2.92 (2.72)	0.001
Well being	253	38.79 (18.34)	3.60 (2.21)	0.001
Total	263	38.79 (18.34)	20.01 (15.7)	0.001

At admission, 197 patients were receiving a breakthrough pain medication (in a rank order: n. 56 non-opioid analgesics, n.51 transmucosal fentanyl products, n.30 oral morphine, and others). At discharge, 247 patients were receiving a breakthrough pain medication, principally represented by opioids (in a rank order: n.83 oral morphine, n.76 transmucosal fentanyl products, and others). The difference was significant (p <0.0005).

### Re-Admissions

Seventy-five patients (23.9%) were readmitted, being 42 (13.4%), 15 (4.8%), and 9 (2.9%) patients previously admitted once, twice, and three times, respectively. Some patients had been admitted several times before (5–20 admissions) in a range of 4–24 months. The reasons for readmission were pain (72 patients) or/and opioid-related effects (11 patients), chemotherapy-related toxicity (11 patients), symptom control (34 patients), end of life care (2 patients). The mean number of days from the last admission was 71 days (SD 171).

### Follow-Up at One Month after Discharge

14 patients (4.5%) were lost at follow up (after 3 phone attempts). One patient or the relative (0.3%) refused the interview. Information regarding the oncologic treatment was available for 215 (68.5%) patients. 94 (29.9%) patients were continuing anticancer treatments, 83 (26.4%) were off-therapy, while in 38 (13.2%) patients the decision was uncertain.

Information regarding the setting of care was unavailable for 100 (31.8%) patients. 122 (38.9%) patients were at home, 62 (19.7%) patients were receiving palliative home care, 5 (1.6%) patients were in hospice. For 25 (8%) patients information was imprecise.

215 patients (68.5%) were still living, 59 patients (18.8%) died at home, two patients died in hospice (0.6%), two patients died in another hospital (0.6%), two patients (0.6%) died in an intensive care unit.

## Discussion

The admission to a specialized APSCU resulted in a decrease in the number of patients continuing an anticancer treatment. This corresponds to a clear change in direction of the clinical trajectory. Moreover, the number of patients who were deemed to continue palliative care at home or in hospice increased, allowing them to be cared in a more appropriate setting. Of interest, hospital stay and mortality rate was extremely low, confirming the specificity of the APSCU. Finally, a dramatic improvement in symptom intensity as well toxicity from oncologic treatments was achieved in a short period of time. Taken together these data suggest the an APSCU admission is not only useful for symptom control or toxicities in complex clinical conditions, but also to allow an appropriate patients’ assessment according to more strict palliative care criteria and a multidisciplinary evaluation of the therapeutic options for individuals. Moreover, some patients may be more appropriately referred to territory resources including home palliative care or hospice. In other words, an APSCU may be a cross-road for advanced cancer patients, switching the lights for the right moment to turn their way.

Many studies have reported the advantages of early palliative care when integrating oncologic treatment, advocating a better quality of life and a longer survival [29–31].However, in these clinical trials, both best supportive care arm and control-arm were poorly defined. As a consequence the risk of over-estimating the effect is high. Moreover, none of these studies documented evidence-based symptom management or modalities of access to palliative care services [32].Regardless of the difficulties in finding an evidence of such a complex issue, oncological departments are the ideal setting to provide multidisciplinary and simultaneous care during all the phases of disease. It is of paramount importance that palliative care starts where the patients are, rather than in other settings where only end-of-life care can be offered for just a few weeks. Some aspects of oncology services deserve particular attention. For example, an outpatient clinic for managing unplanned presentations of cancer patients with drug-induced toxicity and cancer symptoms, provided an improvement of quality of oncology services, avoiding inappropriate admissions and interferences with the ordinary work-plan [2].

Relevant issues relative to the impact of a APSCU in an oncologic department have been recently raised [11–13,15]. It has been described as hospital palliative care may increase the use of palliative care services and the likelihood of dying at home, rather than in hospital [33].Our findings also suggest both a cost and quality incentive for oncological departments to develop APSCU [14,22]. A specialized integrated supportive care team working within the oncology unit may contribute to inpatient costs reduction [34].

At present, APSCUs are available only in a some medical centres in USA [11,15,20]. but not in Europe, where the traditional hospice based approach, inherited from UK tradition, prevails. Even in these centres, the intervals between advanced disease and death, advanced disease and palliative care referral, and palliative care referral and death, were on average 9.4, 5.6, and 1.9 months, respectively [10]More recently, in a multinational European study it has been reported that transitions of care occur late, with 17–27% patients starting palliative care in the last week of life. The majority of patients had severe symptom distress in the last week of life and 33% of patients died in hospital. This finding indicates that further integration of palliative care into oncology care is required in most countries [35].Numerous studies suggest that inpatient units improve symptoms, reduce hospital costs, coordinates care, and reduces inappropriate hospital admissions [36].

There are some limitations regarding the interpretation of data of the present study. This was a single-centre experience and could not reproduced extensively. However, this model, together with those of other North American institutions which have largely provided their data [12]. could be useful for a possible propagation in oncological departments. There is an increasing number of centers of integrated oncology and palliative care, which could potentially provide comprehensive services in supportive and palliative care as part of their routine care. Another limitation is represented by the lack of a control arm. As reported in a recent review, it is quite difficult to select a controlled arm in this population, also from an ethical point of view.

In conclusion, data of this study suggests that the presence of APSCU in a comprehensive cancer center may have a relevant role for managing the therapeutic trajectory of advanced cancer patients, limiting the risk of futile and aggressive treatment while providing an appropriate care setting. Moreover, a broad and comprehensive intervention including the management of cancer–related complex symptoms and toxicities due to oncologic treatments, as well end of life care issues, may improve patients’ care with a short hospital stay and consequently reduced costs. The American Society of Clinical Oncology has progressively increased the visibility of palliative care and has developed education tools to improve oncologist skills in palliative care to facilitate the integration of both processes simultaneously, rather than in a sequential way, just confining palliative care at the end of life. These attitudes may also reduce aggressiveness of cancer care near the end of life [1].The approach presented in this study suggests that the most effective way to improve the care of cancer patients would be to develop formal structures within each oncological department to provide a standardized and integrated approach. The knowledge of these findings would be useful to national policy makers to modify former and old-fashioned policies, to optimize the economic resources, and to improve patients’ care.
